# Supplementary material for: Crystallization Behavior of Poly(ε-Caprolactone)-Hollow Glass Microspheres Composites for Rotational Molding Technology
Source: Polymers (Basel). 2022 Oct 14;14(20):4326. doi: 10.3390/polym14204326 (PMC9609941; doi:10.3390/polym14204326)
Supplement: Supplementary file 1 [file polymers-14-04326-s001.zip › polymers-1918716-supplementary.pdf]

Supplementary materials

Crystallization behavior of poly(ε-caprolactone)-hollow glass microspheres composites for rotational molding technology

Adriano Vignali [1,*], Roberto Utzeri [2], Maurizio Canetti [1] and Fabio Bertini [1]

[1] Istituto di Scienze e Tecnologie Chimiche "Giulio Natta" (SCITEC) – CNR, Via A. Corti 12, 20133 Milano, Italy
[2] Istituto di Scienze e Tecnologie Chimiche "Giulio Natta" (SCITEC) – CNR, Via De Marini 6, 16149 Genova, Italy
* Correspondence: adriano.vignali@scitec.cnr.it

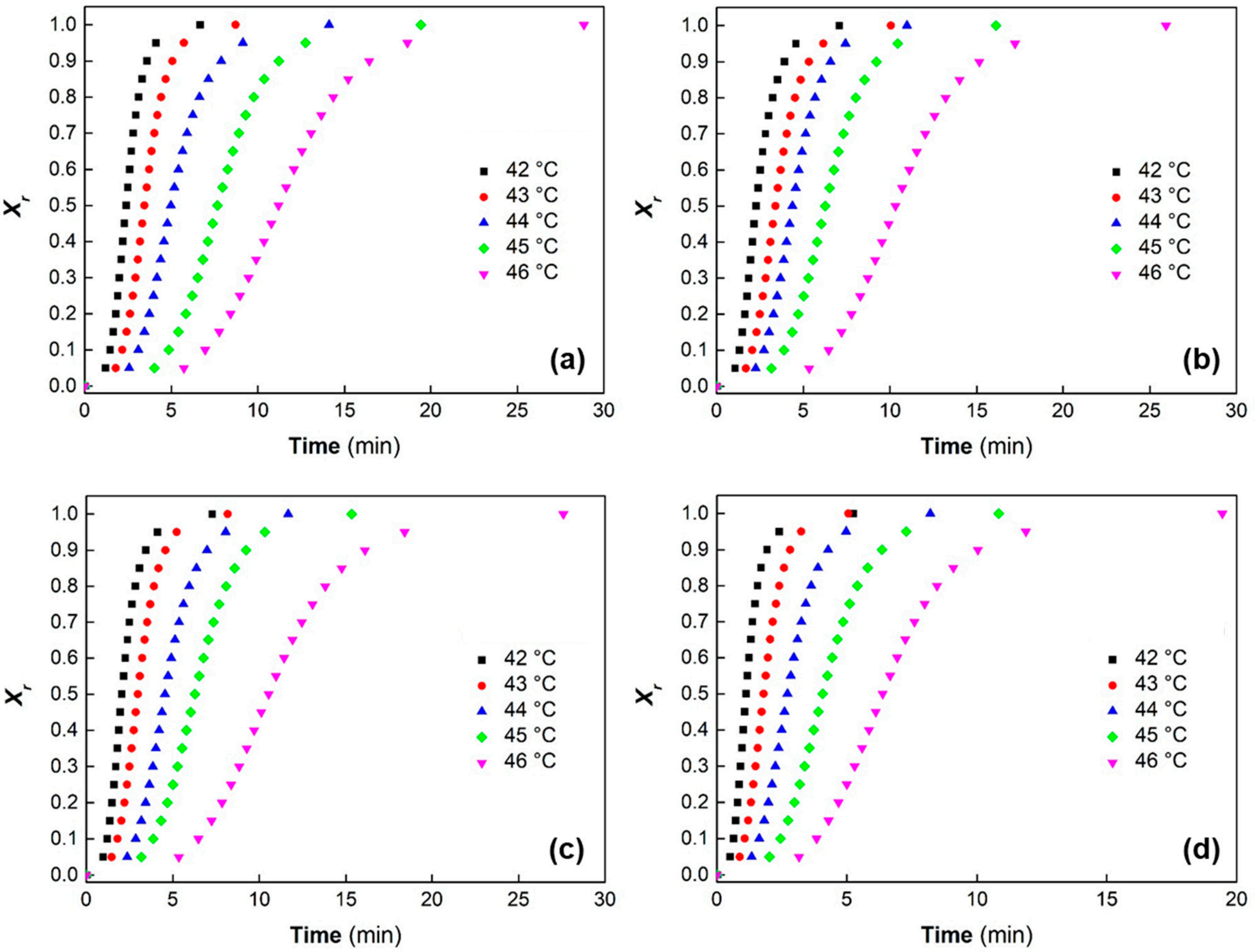

Figure S1. Evolution of relative crystallinity as a function of time for PCL-HGM15 (**a**), PCL-HGM20 (**b**), PCL-HGM25 (**c**) and PCL-HGMf20 (**d**) under isothermal conditions at different T_c.

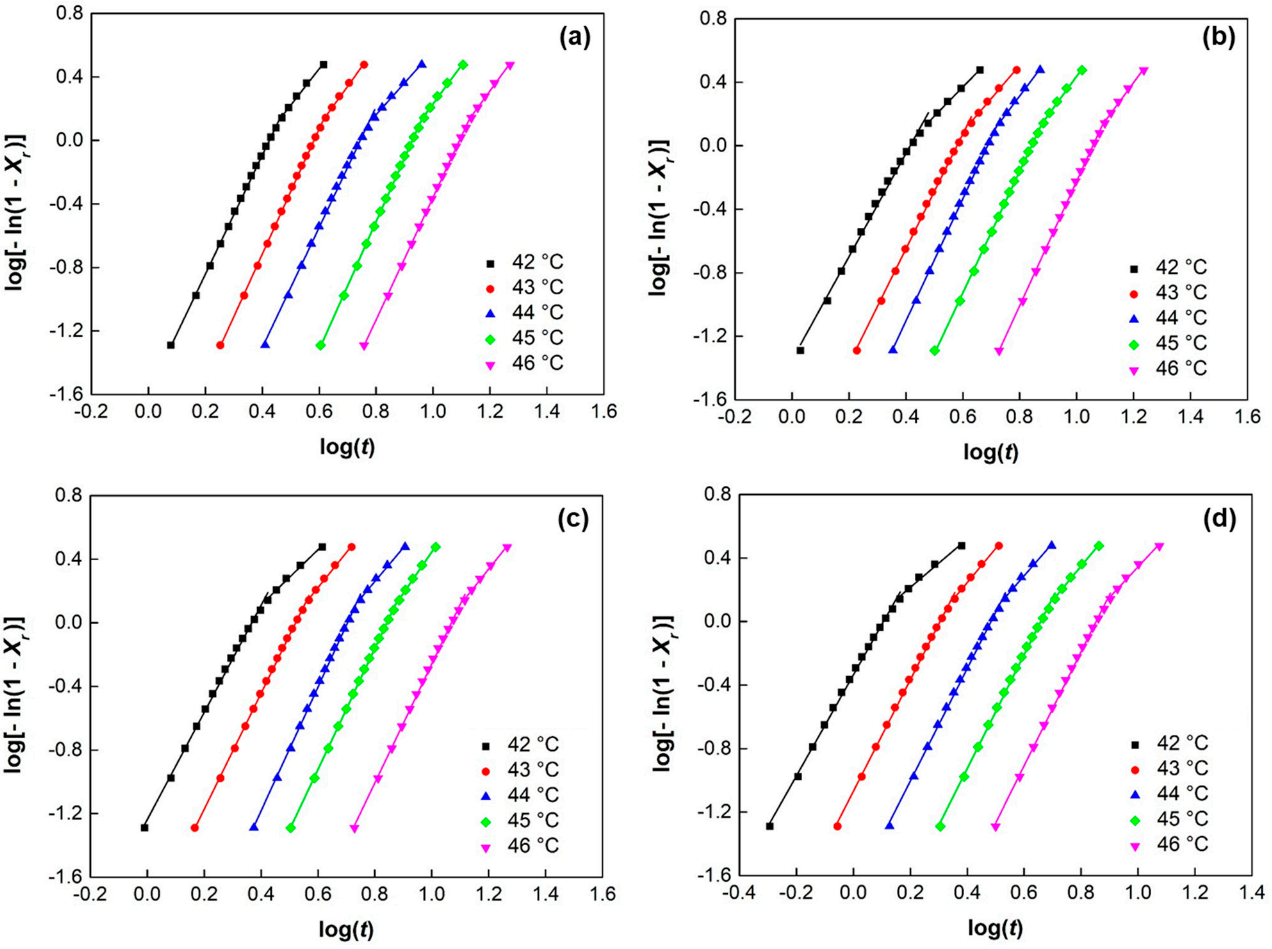

Figure S2. Plots of log[– ln(1 – X_r)] vs. log t for isothermal crystallization of PCL-HGM15 (**a**), PCL-HGM20 (**b**), PCL-HGM25 (**c**) and PCL-HGMf20 (**d**) at different T_c.
